# Characterization of a Halotolerant Fungus from a Marine Sponge

**DOI:** 10.1155/2019/3456164

**Published:** 2019-11-23

**Authors:** Yitayal S. Anteneh, Melissa H. Brown, Christopher M. M. Franco

**Affiliations:** ^1^College of Medicine and Public Health, Medical Biotechnology, Flinders University, Bedford Park, Adelaide, SA 5042, Australia; ^2^Department of Medical Microbiology, College of Medicine, Addis Ababa University, Addis Ababa, Ethiopia; ^3^College of Science and Engineering, Flinders University, Bedford Park, Adelaide, SA 5042, Australia

## Abstract

**Introduction:**

Marine sponges have established symbiotic interactions with a large number of microorganisms including fungi. Most of the studies so far have focussed on the characterization of sponge-associated bacteria and archaea with only a few reports on sponge-associated fungi. During the isolation and characterization of bacteria from marine sponges of South Australia, we observed multiple types of fungi. One isolate in particular was selected for further investigation due to its unusually large size and being chromogenic. Here, we report on the investigations on the physical, morphological, chemical, and genotypic properties of this yeast-like fungus.

**Methods and Materials:**

Sponge samples were collected from South Australian marine environments, and microbes were isolated using different isolation media under various incubation conditions. Microbial isolates were identified on the basis of morphology, staining characteristics, and their 16S rRNA or ITS/28S rRNA gene sequences.

**Results:**

Twelve types of yeast and fungal isolates were detected together with other bacteria and one of these fungi measured up to 35 *μ*m in diameter with a unique chromogen compared to other fungi. Depending on the medium type, this unique fungal isolate appeared as yeast-like fungi with different morphological forms. The isolate can ferment and assimilate nearly all of the tested carbohydrates. Furthermore, it tolerated a high concentration of salt (up to 25%) and a range of pH and temperature. ITS and 28S rRNA gene sequencing revealed a sequence similarity of 93% and 98%, respectively, with the closest genera of *Eupenidiella*, *Hortaea,* and *Stenella*.

**Conclusions:**

On the basis of its peculiar morphology, size, and genetic data, this yeast-like fungus possibly constitutes a new genus and the name *Magnuscella marinae*, gen nov., sp. nov., is proposed. This study is the first of its kind for the complete characterization of a yeast-like fungus from marine sponges. This novel isolate developed a symbiotic interaction with living hosts, which was not observed with other reported closest genera (they exist in a saprophytic relationship). The observed unique size and morphology may favour this new isolate to establish symbiotic interactions with living hosts.

## 1. Introduction

Fungi contribute a large share of the microbial community on earth [1] and participate largely in organic matter decomposition, nutrient recycling, and symbiotic interactions with other living forms [2]. About 1.5 million species of fungi are distributed worldwide, and most of the current knowledge about them has originated from cultivable representatives, primarily from terrestrial environments [3, 4]. Unlike their terrestrial counterparts, little is known about the diversity of the fungal community in the marine environment [5]. In marine environments, several families of fungi exist, which contribute about 0.6% of the total fungal community in the world [6, 7]. There have been attempts to isolate fungi from different marine habitats including seawater [8], sea sediments [9], and very hypoxic deeper parts of the oceans [10], where these species have been in association with different marine plants and animals such as algae [11], corals [12], and sea fans [13].

In addition to the above-mentioned habitats, marine sponges can also provide a home for fungi. About 50% of the mass of marine sponges is due to microorganisms which is many times higher compared to the numbers found in seawater [14]. These sponges have established close associations with a range of prokaryotic and eukaryotic microbes [15–23]. So far, most studies have focussed on characterization of sponge-associated bacteria and archaea [17, 23]. In contrast, information about sponge-associated fungi is very limited [22]. Fungi are ubiquitous and it is relatively easy to isolate them from the inner tissue of the sponges [14, 24]. However, most studies of fungal isolation from sponges have an emphasis on detection and characterization of the compounds they produce as fungi are a major producer of novel marine metabolites [14, 25–27].

Hundreds of fungal strains, representing three phyla of Ascomycota, Zygomycota, and Mitosporic fungi, have been isolated from marine sponges [26, 28, 29]. However, those studies mostly characterized fungi based on their gene profile, morphology, physical properties, or chemical characteristics [30, 31], and no single study has attempted the complete characterization of fungal isolates from sponges.

While studying the diversity of bacteria in marine sponges of South Australia, 12 types of fungal strains were detected. One of these presented a characteristic yeast-like and filamentous appearance, large size, and unique colour compared to the rest of the group. This study addressed the morphological, physical, chemical, and genotypic properties of this yeast-like fungus. As far as we know, this is the first study to report the complete characterization of a sponge-associated yeast-like fungus from the marine environment of South Australia.

## 2. Materials and Methods

### 2.1. Sponge Sample Collection, Processing, and Isolation

Sponge samples were collected from multiple South Australian marine environments. These included sites at Glenelg (34° 58′ 406″S, 138° 30′ 494″E) and Rapid Bay (35.5229°S, 138.1854°E) at a depth of 6–10 m and a water temperature of 15°C at the time of collection. The samples were placed in zip lock plastic bags containing fresh seawater and transported to the laboratory on ice. In the laboratory, sponge samples were maintained in an aquarium system. Sponge samples were processed and microbes were isolated following previous protocols [32–35]. In brief, all samples were placed in sterile seawater to remove any external organic matter followed by surface sterilization with 70% ethanol and drying them in a sterile laminar flow. Approximately 1 cm^3^ of dried sponge pieces was removed and homogenized with 10 volumes of sterile seawater using a sterile pestle and mortar. A 10-fold dilution series (10^−1^ to 10^−6^) was prepared and 100 *μ*l of the three highest dilutions was inoculated onto the following media, prepared in sterilized natural seawater: soluble starch yeast extract peptone agar (SYP), asparagine peptone agar (APA), natural seawater agar (SWA), humic acid vitamin agar (HV), nutrient agar (NA), marine agar (MA), and tryptone soya agar (TSA) in six replicates and incubated at 27°C in the dark for two months.

### 2.2. Purification of Isolates

Isolation plates were checked at least once a week and microbial colonies that emerged were picked individually, streaked onto fresh medium, and incubated again at 27°C for 2 weeks. Vials of purified microorganisms were stored in sterile 50% (w/v) glycerol at −20°C [31].

### 2.3. Morphological and Molecular Identification

The existence of yeast-like fungi was identified primarily by microscopic observation of the yeast form using wet mount and lacto phenol cotton blue stain preparations [36]. Further morphological identification was attained by growth on standard fungal media of malt extract agar (MEA) [37], cornmeal agar (CMA) [38], potato dextrose agar (PDA) [30, 39], and Sabouraud dextrose agar (SDA) [40]. All identification media were supplied by Oxoid. Colony shape, size, colour and nature of hyphae, growth rate, and features of conidia were the features considered for morphological identification.

Sequence based phylogenetic analysis was employed for molecular identification of the isolates. DNA was extracted using a cetyltrimethylammonium bromide (CTAB) method [41]. The 28S rRNA gene was amplified and sequenced using LROR (ACCCGCTGAACTTAAGC) and LR5 (TCCTGAGGGAAACTTCG) primers. Similarly, the internal transcribed spacer gene (ITS) was amplified and sequenced using the primer set ITS3 (GCATCGATGAAGAACGCAGC) and ITS4 (TCCTCCGCTTATTGATATGC) [42]. All amplification reactions were carried out in a Swift Thermal Cycler (Esco GB Ltd.), with reaction cycles of 95°C for 10 min, 35 cycles of 94°C for 1 min, 52°C for 1 min, and 72°C for 2 min, followed by a cycle of 72^o^C for 10 min, and 12°C cooling. The PCR products were detected by electrophoresis on 1% (w/v) agarose gels and, following cleaning of the products, were sequenced at Macrogen, South Korea. The nucleotide sequences were compared with the GenBank (NCBI and EMBL) database using BLASTN (https://blast.ncbi.nlm.nih.gov/Blast.cgi). Sequences were initially aligned using the multiple alignment program CLUSTAL W version 2.0 [43], and phylogenetic trees were constructed using the neighbor-joining method (based on 1000 bootstrap iterations) with MEGA version 7 [44].

### 2.4. Physical Characterization

Temperature, pH, and NaCl tolerance of the isolates were examined using PDA medium. A 0.5 cm diameter agar block was cut from a three-day-old culture grown on PDA and inoculated onto freshly prepared PDA medium for the following assays. Plates for the temperature tolerance test were incubated at 5°C, 15°C, 20°C, 25°C, 30°C, and 37°C for 3 weeks, while those for the acid tolerance (pH of 4, 5, 6, 7, 8, and 9) and NaCl tolerance (1, 2, 3, 4, 5, 10, 15, 25, and 30% NaCl) were incubated at 27°C for 3 weeks [30]. Each treatment was carried out in quadruplicate and the mean diameter of the colony growth was used for data analysis.

### 2.5. Biochemical Characterization

#### 2.5.1. Carbohydrate Assimilation

Growth on the following D-carbohydrates was assessed: inositol, glucose, maltose, mannose, sucrose, fructose, melezitose, trehalose, raffinose, cellobiose, lactose, and xylose. A 20% solution of each carbohydrate was prepared in a 10x concentration of yeast nitrogen base (Difco) and filter sterilized (Minisart®, 0.22 *μ*m) [45]. Basal agar medium was prepared by dissolving 6.7 g of Bacto Yeast Nitrogen Base and 20 g of high-grade agar in 1 litre of RO water. A 24–48 h old culture was used to prepare a yeast suspension in 2 ml MilliQ water and mixed with 18 ml of molten agar and placed into a 90 mm Petri plate. Following solidification, 50 *μ*l of each carbohydrate was placed into 12 wells (6 mm diameter) and the plate was incubated for three weeks. Dense growth around the well was assigned as positive for assimilation of the particular sugar [46].

#### 2.5.2. Carbohydrate Fermentation

The following D-carbohydrates were tested; glucose, maltose, sucrose, trehalose, lactose, and galactose with a basal inorganic nitrogen medium containing, per litre, (NH_4_)_2_HPO_4_ 1 g, KCl 0.2 g, MgSO_4_.7H_2_O 0.2 g, and agar 15 g. Fifteen millilitres of 0.04% bromocresol purple medium was added, per litre, as a pH indicator. After autoclaving, the medium was transferred to sterilized 10 ml tubes and filter sterilized carbohydrate was added to a final concentration of 1%. A 24–48 h old culture grown in PDA medium was streaked onto agar tubes with carbohydrates and with no carbohydrates as negative control. For each carbohydrate, the experiment was designed in duplicate and the tubes were incubated at 27°C for 2 weeks. Change in colour of the medium to yellow was taken as a positive indication of carbohydrate fermentation [47, 48].

## 3. Results and Discussion

### 3.1. Morphological Assessment

**Figure 1:**
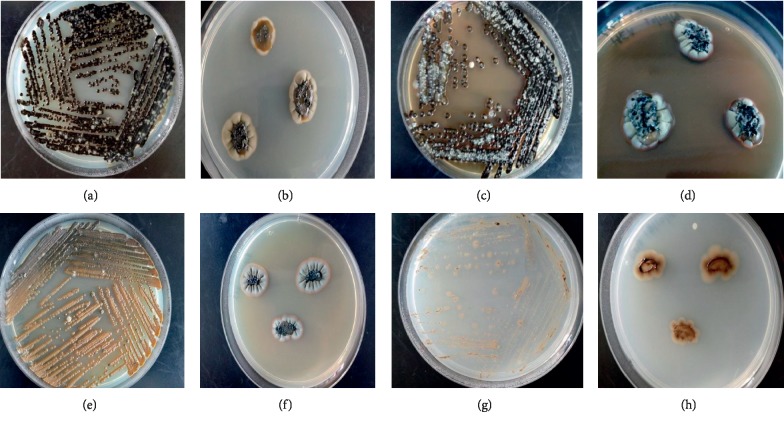
Morphological characteristics of front and back views of the isolate grown on four different media. (a, b) Potato dextrose agar (PDA): yeast forms were globose, shiny, dark green turning to black, produced flat greenish grey spores surrounding green centre. (c, d) Malt extract agar (MEA): yeast forms were globose, shiny, black, produced raised light green spores. (e, f) Sabouraud dextrose agar (SDA): yeast forms were globose, not shiny, light brown and turned from dark brown to black, produced raised grey spores with black centre. (g, h) Corn meal agar (CMA): yeast forms grew poorly, with light brown colour, no spores.

This study was biased towards the isolation of bacteria, including Actinobacteria, from sponge samples obtained from Rapid Bay and Glenelg. Filamentous fungi which emerged despite the use of cycloheximide (an antifungal) were not picked up but yeast with morphologies similar to bacteria would have been. The novel yeast-like fungi, together with another 11 yeasts, were identified by microscopy. These 12 fungi accounted for 1% of the total microbial isolates in the study and were isolated from three sponge samples (RB 16, RB17, and RB18), collected in the same site. The new taxon was isolated from sponge sample RB16. These yeasts were isolated from growth on SYP (six yeasts including the new taxon presented here), APA (four yeasts), and MA (two yeasts). During the microscopic evaluation, one of the yeasts, a yeast-like fungus from a pink unidentified marine sponge from Rapid Bay, South Australia, was noted to have a large diameter and was selected for a complete phenotypic and genotypic characterizations. The morphological characteristics of this fungus were assessed by culture onto four common fungal identification media. Each preparation grew very slowly on all four media with an average colony diameter of 16 mm after 20 days of incubation. During this initial period, the colonies appeared as yeast (wet, nonfilamentous); thereafter the colonies advanced to dry and moldy forms. As indicated in Figure 1, the nature of the colonies varied depending on the media composition. Pronounced colony growth was observed in all media with the exception of those cultured onto CMA. On PDA, MEA, and SDA media, significant variations were observed in the appearance of the colonies, in particular, the front view of the colonies.

The front and reverse views of the colonies are among the many factors considered for standard fungal identification. At the early stage of growth, in all media, the colonies were smooth, wet, and pale brown to black in colour. Gradually, with the exception of those growing on CMA, the colonies developed aerial mycelium and appeared as green, brown, and black filamentous fungi. This type of incubation period dependent morphological dimorphism is not reported in studies describing fungi from marine sponges [39, 47] or other sources [30]. This form of transformation is not the same as temperature-dependent fungal dimorphism which is commonly observed in some fungal species [49]. This is an important evolutionary observation of the isolate, and further study is required to understand the mechanisms behind this morphological variation and the specific role of morphological forms in fungal biology and their interaction with sponges.

**Figure 2:**
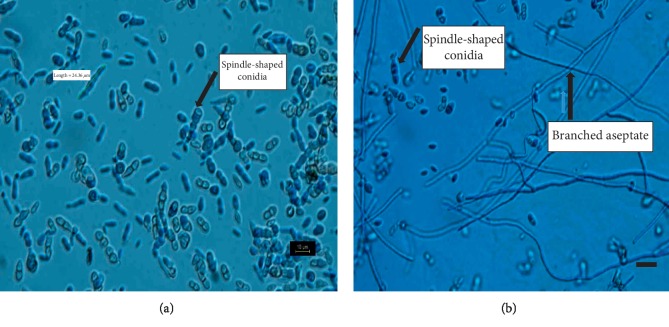
Morphological characteristics of the *Magnuscella marinae* cells after 3 weeks of incubation. (a) Lactophenol cotton blue preparation showing yeast-like colonies; (b) preparation from mycelium under 40x magnification and the scale bar of 10 *μ*m.

Lactophenol cotton blue preparations from the yeast-like form revealed elliptical conidia occurring in single, bicellular, and short chains of spindle-shaped blastospores (Figure 2). Their size varied from 6 to 35 *μ*m in diameter. Similar preparations from mycelia showed branched, thick-walled, smooth, and aseptate hyphae with aggregated mass of conidia. Conidiogenous cells were observed within the hyphae forming chains of conidia. Compared to related fungal strains, this isolate displayed unique features including aseptate hyphae, spindle-shaped conidia, and a large size of the yeast form [30, 50–52]. Phenotypic observations of morphological forms such as the nature of hyphae and conidia are the most commonly considered characteristics for Ascomycota taxonomy—the fungal phylum to which this isolate belongs [53].

### 3.2. Phylogenetic Analysis

**Figure 3:**
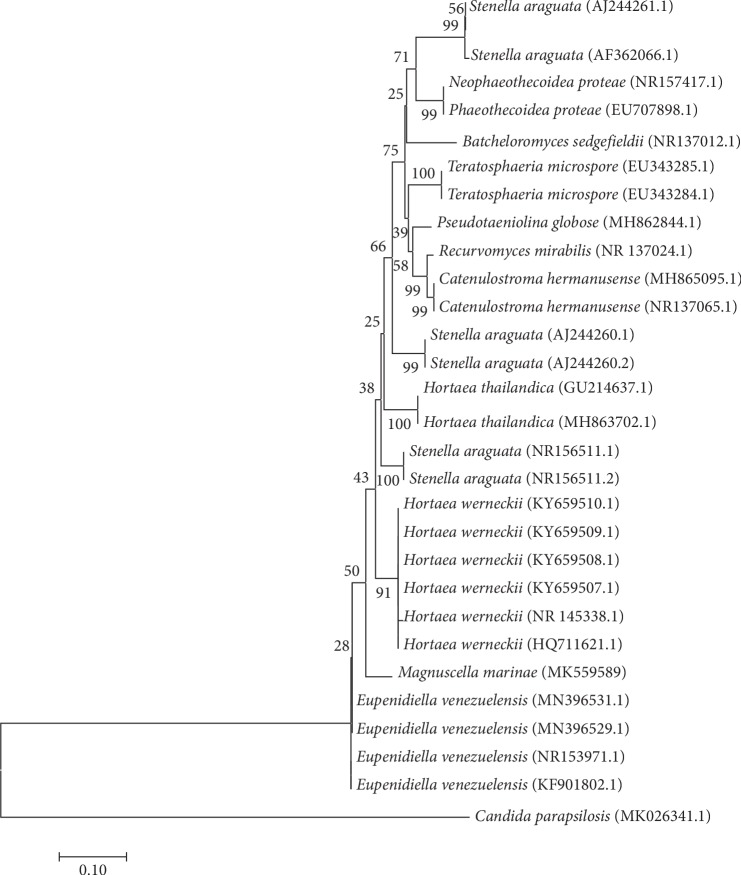
Phylogenetic analysis of the *Magnuscella marinae* inferred from ITS region gene. Evolutionary relationships were assessed using the neighbor-joining method [47], and bootstrap values (>50%) are shown above the branch. *Candida parapsilosis* was used as an out group. Bar 10% sequence divergence.

**Figure 4:**
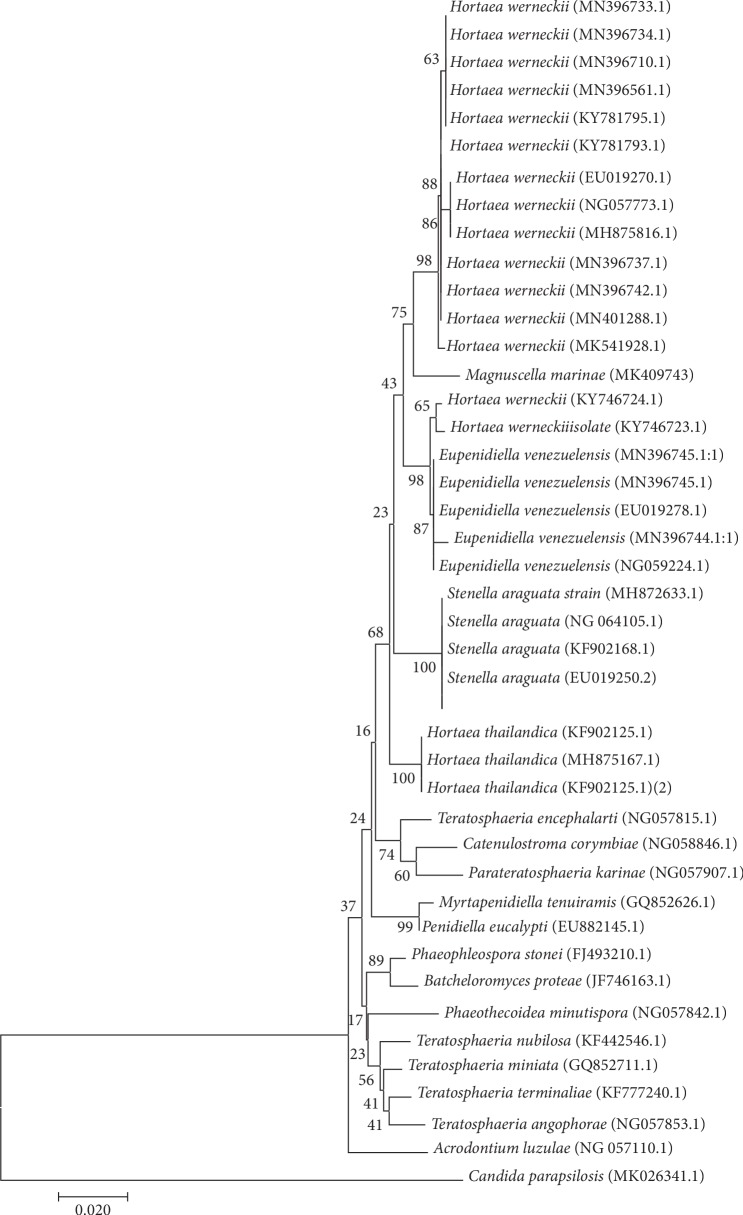
Phylogenetic analysis of the *Magnuscella marinae* inferred from 28S rRNA gene. Evolutionary relationships were assessed using the neighbor-joining method [47], and bootstrap values (>50%) are shown above the branch. *Candida parapsilosis* was used as an out group. Bar 2% sequence variation.

Genetic identification of the isolate was achieved through sequencing of the ITS region (MK55958) and the 28S rRNA gene (MK409743). BLASTN analysis of the ITS sequence revealed the most closely related strains were *Eupenidiella venezuelensis* (93%), *Hortaea thailandica* (89%), and *Stenella araguta* (89%), which was supported by the phylogenetic tree (Figure 3). In the same manner, a BLASTN of the 28S rRNA gene sequence confirmed the close relationship with the same species but with sequence similarities of 98%, 97%, and 96.5%, respectively, with these three species, which was supported by the phylogenetic tree (Figure 4). As these data were also supported with the genetic data, we assigned the name *Magnuscella marinae* gen. nov., sp. nov., (meaning large (Latin) and Cell (English), with the species named *marinae*, denoting the source which was a marine sponge).

The fungal species which are most closely related to *Magnuscella marinae* belong to phylum Ascomycota, class Dothideomycetes, order Capnodiales, and family either Teratosphaeria (such as genus *Eupenidiella and Hortaea*) [30, 54] or Mycosphaerellaceae (such as genus *Stenella*) [55]. These two families are generally characterized by their widespread presence as saprophytes, opportunistic human pathogens, and phytopathogens [51, 54]. The filamentous form produced branched, septate, and chromogenic hyphae, which measure about 2–6 *μ*m in diameter [52]. Conidiogenous cells, which exist as integral or terminal parts of the hyphae, appear as subcylindrical or slightly swollen tips [30, 54]. It is important to determine whether this new genus is pathogenic to mammals or plants given its taxonomic relatedness to known pathogens. Neither this species nor the most common phylogenetically related species to this isolate have been reported from marine sponges, as most have been obtained from the terrestrial environment and plants.

### 3.3. Physical Characterization

**Figure 5:**
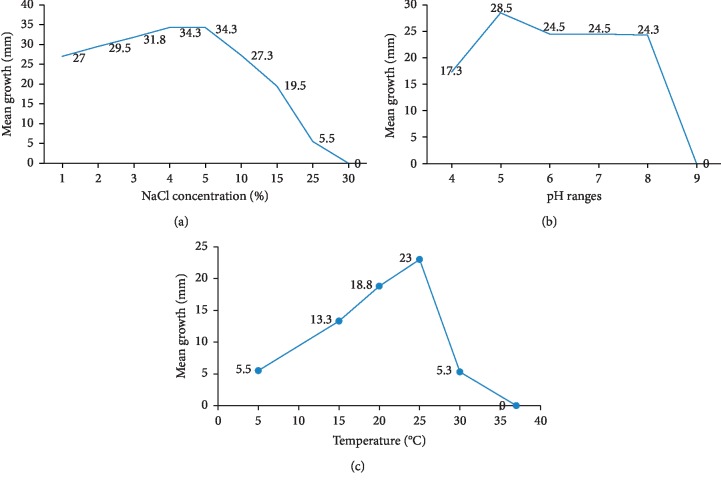
Growth of *Magnuscella marinae* after 3 weeks incubation with variation in (a) NaCl concentration, (b) pH, and (c) temperature.

The yeast-like fungus *Magnuscella marinae* was capable of growing at temperatures ranging from 5 to 30°C, with optimum growth seen at 25°C (Figure 5(c)). At lower and higher temperatures, the fungus grew slowly with limited hyphae or mycelium production. The fungus grew well within pH ranges between 4 and 8, with somewhat higher than average growth observed at pH 5 (Figure 5(b)). The fungus tolerated up to 25% NaCl (Figure 5(a)). It was also observed that growth was highly restricted in medium devoid of NaCl (data not presented) indicating that the fungus requires some degree of salt to grow, a trait common to marine microorganisms. The observed survival under extreme environmental conditions might explain their activity in the highly fluctuating marine environments, including within sponges.

Similar to our findings, some related species in genus *Hortaea* are known for their ability to tolerate a wide range of temperature, pH, and salt concentrations. These species have been widely studied to reveal the mechanisms behind their survival under harsh environmental conditions. A number of unique pathways have been identified that could attribute for this adaptation [30]. Similar to the members of the genus *Hortaea*, *Magnuscella marinae* can be used as a potential model organism to assess the mechanisms behind survival in different harsh environmental conditions including high salt concentrations.

### 3.4. Biochemical Characteristics

As indicated in Table 1, the isolate displayed a capability of fermenting almost all the tested carbohydrates with the exception of lactose. In addition, they were able to utilize about two-thirds of the tested sugars. No data are available in the literature regarding carbohydrate assimilation and fermentation profiles for the closest phylogenetically related species. The findings of this study, therefore, could form the basis for a comprehensive comparative study of related fungi. The ability of *Magnuscella marinae* to assimilate and ferment several carbohydrates may make them a good candidate organisms for the production of lipids, proteins, or antimicrobial compounds via industrial-scale fermentation, if they do not possess pathogenic determinants [56].

Since the sponge environment is quite unique, it is likely that *Magnuscella marinae* has adapted to form a relationship with its host and other microorganisms present in the sponge tissue. Their ability to utilize a range of carbohydrates might help them to survive in a competitive environment like sponges, as their metabolic diversity provides for the uptake of an alternative nutrient source in the absence of others. Apart from their importance to fungi, this metabolic diversity together with their large size could make *Magnuscella marinae* as a potential biological catalyst or producer of feedstock in the fermentation industry. Furthermore, the isolate could be a model organism to study the effect of cell size on the efficiency of cellular processes and whether a large cell size offers any advantages for bioprocessing or as a host for recombinant protein production.

## 4. Conclusions

The fungi constituted about 1% of the total “bacteria-like colonies” isolated in this study, indicating their existence at very low levels in marine sponges. Since none of the closely related species have been reported from marine sponges, it is unusual to find the largest reported fungus residing within a marine sponge. However, this new taxon isolate may have evolved within a living macroorganism. This indicates that the symbiosis between fungus and sponge appears to function well and it would be worth looking for other members of this new fungus genus in the same and related sponge species. Furthermore, the unusually big size and metabolic versatility of the new isolate could be a significant evolutionary phenomenon which contributes to their existence in a competitive environment like sponges.

This is an important evolutionary observation as symbiotic “mycorrhizal” fungi are known to form strong interactions within terrestrial plants. This new taxon widens the existing knowledge on the ecology of marine fungi. It will create an opportunity to investigate the potential factors that provide the evolutionary changes which favour their survival within harsh environmental conditions such as high salt.

This study could be the base line for future comparative studies. Marked genotypic variations accompanied with unique properties such as large size, aseptate hypha, and spindle-shaped conidia compared to the most closely related species, the isolate was considered to be a candidate for a novel genus.

## Figures and Tables

**Table 1:** D-carbohydrate fermentation and assimilation pattern of the *Magnuscella marinae* isolate.

Carbohydrates	Fermentation	Assimilation
Glucose	✓	✓
Maltose	✓	✓
Sucrose	✓	✓
Trehalose	✓	✓
Lactose	X	X
Galactose	✓	ND
Inositol	ND	X
Mannose	ND	✓
Fructose	ND	X
Melezitose	ND	✓
Raffinose	ND	✓
Cellobiose	ND	X
Xylose	ND	✓

ND: not done.

## Data Availability

The data used to support the findings of this study are included within the article.
